# People with disabilities and income-related social protection measures in South Africa: Where is the gap?

**DOI:** 10.4102/ajod.v6i0.300

**Published:** 2017-09-26

**Authors:** Jill Hanass-Hancock, Tamlyn C. McKenzie

**Affiliations:** 1 SA Medical Research Council, South Africa; 2 School of Health Science, University of KwaZulu-Natal, South Africa; 3 School of Accounting, Finance and Economics, University of KwaZulu-Natal, South Africa

## Abstract

**Background:**

People with disabilities are at increased risk of poverty, particularly in low-and middle-income countries. However, recent evidence suggests that this association is more nuanced than previously anticipated and that we need better data to understand the opportunity and out-of-pocket costs that diverse groups of people with disabilities may experience.

**Objective:**

This paper discusses if disability is associated with opportunity cost and loss of income both on the individual and household level in South Africa, and if these costs differ depending on disability type and severity.

**Methods:**

For this purpose, the paper analyses General Household Survey 2011 data (people between 15 and 59) using descriptive statistics disaggregated via disability type and severity. The paper also assesses if social grants counteract these costs and reduce economic vulnerability.

**Results:**

The analysis of the data reveals that people with disabilities are affected by issues relating to multidimensional poverty such as lower educational attainment and fewer employment opportunities. In addition, households of people with disabilities (with the exception of milder visual problems) earn significantly less than households without people with disabilities, and this particularly applies to households with people with severe disabilities. This vulnerability also varies by disability type. The country’s social protection mechanisms, in terms of social grants, counteract economic vulnerability to some extent but do not consider the nuanced economic impact of diverse conditions nor the increased out-of-pocket costs related to disability.

**Conclusions:**

This calls for more equitable social protection mechanisms that include accessible services, livelihood programmes and disability benefits.

## Introduction

It has been estimated that one billion people or 15% of the population worldwide have one or more disabilities and that this number is increasing, particularly in resource-poor settings (World Health Organisation & World Bank 2011). Disability and poverty are understood as being interlinked in a vicious cycle with disability increasing the risk of poverty and poverty leading to disability. In the past two decades, researchers have argued that this particularly applies to low-and middle-income countries (LMIC) (Eide & Loeb 2006; Elwan 1999; Hanass-Hancock & Mitra 2016; Mitra, Posarac & Vick 2011; Mont & Nguyen 2011). Not surprisingly, there has been a call for disability-related issues to be factored into poverty alleviation programmes and into the emerging social protection mechanisms in these countries (Schneider et al. 2011).

It has been increasingly recognised that marginalised groups such as people with disabilities have been ignored in previous efforts to eradicate poverty and inequality. The 2013 United Nations (UN) report on ‘A new global partnership: Eradicate poverty and transform economies through sustainable development’ acknowledges this oversight in the previous developmental agenda (Millennium Development Goals) (UN 2013a, 2013b). The report shifts focus towards a vision of ‘leaving no one behind’ (UN 2013a) and lists people with disabilities among vulnerable populations who need to be included to achieve sustainable development.

Current literature highlights that the poverty-disability link is more complex and nuanced than previously anticipated (Banks & Polack 2013; Graham et al. 2014; Groce et al. 2011; International Labour Office & International Disability Alliance 2015; South African Department of Social Development 2016). This literature highlights the multidimensional aspect of poverty in the context of disability, highlighting that people with disabilities may experience barriers to health, education and employment (directly reducing opportunities and potential income), as well as disability-related out-of-pocket costs. In addition, the group of people with disabilities is diverse and experiences different barriers and costs depending on their impairment, gender and environmental factors. Little is known on exactly how and which people with disabilities are affected by poverty, and how social protection mechanisms might impact them.

Therefore, more rigorous research on the poverty-disability link is needed. This includes disaggregated data to better understand the nuances of disability and its impact on individuals, households and society at large. This particularly applies to middle-income countries (MICs) such as South Africa that carry the global burden of poverty (IDS 2010) and are also currently developing social protection mechanisms (International Labour Office & International Disability Alliance 2015; South African Department of Social Development 2015). South Africa has a history of social security instruments targeting people with disabilities; therefore, an investigation of the impact of current practice on the economic vulnerability of people with disabilities in this country serves as an example for other MICs.

## Background

Extending social protection programmes to LMIC has become a focus of poverty alleviation strategies in the last decade (Frye 2005; Hagemejer & ILO 2009; Kabeer 2009; International Labour Office & International Disability Alliance 2015). This includes the provision of access to essential health care services, social assistance (e.g. old age, disability and child benefits) and affordable insurance schemes (Hagemejer & ILO 2009). In this context some MICs, including South Africa have introduced cash transfers targeting people with disabilities and these are increasingly becoming the subject of a growing body of research (Booysen 2004; CASE 2005; De Koker, De Waal & Vorster 2006; International Labour Office & International Disability Alliance 2015; Mitra 2005, 2010; Nattrass 2004, 2005; South African Department of Social Development 2016).

**FIGURE 1:**
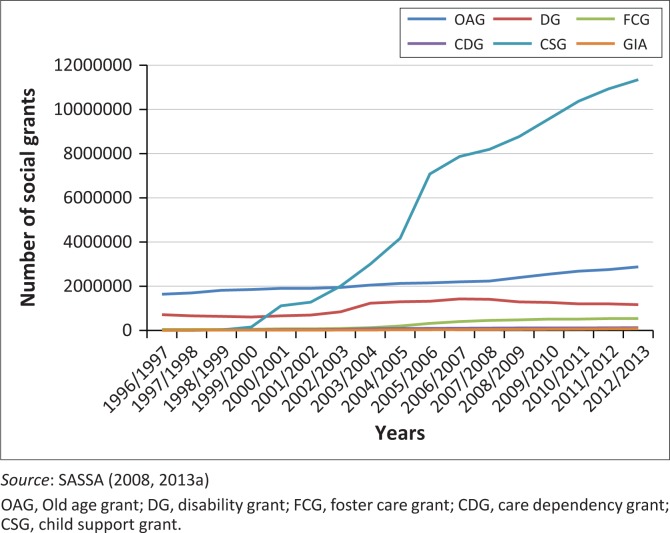
Number of social grants since 1996 in south Africa (excl. War Veterans Grant).

South Africa has undergone a rapid change in social policy and protection mechanisms in the past two decades. Since 1996, the right to social protection is ensured within the South African constitution which states that ‘everyone has the right to access social security, including with appropriate assistance for those who are unable to support themselves and their dependents’ (Section 27, 1C: Constitutional Assembly 1996). The country provides a variety of social security grants (Figure 1) including the Old age grant (OAG), disability grant (DG) (available since 1946), foster care grant (FCG), care dependency grant (CDG), child support grant (CSG) and grant-in-aid (GIA). With the exception of the CDG and GIA, the uptake of grants has increased considerably in the past two decades with the CSG showing the largest increase (Figure 1). All grants are means tested and non-contributory. The government also provides a War Veterans Grant, but this has been excluded from our study because of the relatively small number of recipients and the significant decline in uptake over time.

The DG, CDG and GIA (see Table 1) are social protection mechanisms that directly target people with disabilities and their households. The CDG and GIA are only accessed by a small number of people (SASSA 2008, 2013a). The main grant accessed by households with people with disabilities is the DG. The DG is available to adults with disabilities who earn below a certain threshold (which is adjusted every year). People who are older than 60 years qualify for the OAG and caregivers of children with severe disabilities for the CDG if their income is below the threshold. The country also provides a number of other social protection measures for those who earn below a certain level of income, such as fee-free schools, free essential healthcare and housing subsidies (SASSA 2013b; South African Department of Social Development 2015). South Africa also has progressive labour regulations that favour the employment of previously disadvantaged people such as people with disabilities (Department of Labour South Africa 2011). In addition, people with disabilities who are in the taxable income bracket can claim part of their disability-related costs back through the country’s tax rebate system.

**TABLE 1:** Overview of current social protection mechanisms in South Africa.

Type of benefit	Requirement	Comment
Disability grant (DG)	Means test and disability eligibility	18–59 years old with medical assessment, passes means test (including spouse) and not in a public institution.
Old age grant (OAG)	Means test and age	60 years or older who passes means test (including spouse).
Care dependency grant (CDG)	Means test and disability eligibility	For a child under the age of 18, medical assessment stating child’s severe disability; must pass means test.
Child support grant (CSG)	Means test and for children 0–17	Any parent who passes means test (including spouse).
Foster care grant (FCG)	Fostering children	Any parent who fosters a child.
Grant-in-aid (GIA)	Means test	Recipients of old age, war veterans or disability grant who require full time attendance by another person owing to physical or mental disability.
No-fee schooling	Areas identified as poor	These ‘no-fee’ schools receive all their required funding from government. In 2011 55% of all schools were no-fee schools serving about 43% of all learners in South Africa. (www.southafrica.info/services/education/edufacts.htm).
Free access to basic healthcare	Public healthcare	Basic services for people visiting public primary health facilities.
Free housing	Means test	Reconstruction and Development Programme (RDP houses for poor households without a home.
Gap housing	Means test and profession	A policy that addresses the housing aspirations of people such as nurses, fire fighters, educators and members of the armed forces, who earn between R3000 and R15 000 per month and, therefore, did not qualify for RDP houses and did not earn enough to obtain home loans.
Supported transport	Disability eligibility test	‘Dial-a-ride’ public transport service for people with physical disabilities who, due to the nature of their disability, are unable to board and/or alight from mainstream public transport such as trains, buses and minibus taxis for their daily commute between home and work. This was subsidised in 2014.

*Source:* SASSA (2013/2014)

The DG is a social protection mechanism directed at people with disabilities. Its impact on household income can be examined using relevant South African household surveys. The DG includes a means and assets test as well as an eligibility assessment that confirms disability status (SASSA 2013b). The disability eligibility assessment was originally conducted by a medical officer alone. In 2001, this assessment procedure changed, and more decision-making power was allocated to provinces. As a result, the process differs across provinces with some using medical officers and others using expert panels consisting of are habilitation officer, a representative from a Disabled People’s Organisation (DPO) and a medical officer (Mitra 2010). Some provinces use both the medical officer and the expert panel. Hence, there has been a potential for a diverse interpretation of what constitutes disability with some conditions such as those that are less visible being less likely to receive a grant in some areas, while people with similar conditions may receive a grant in a different area where there is more awareness of less visible or milder forms of disability.

The period 2001–2006 has seen a steady increase in applications for and the uptake of the DG (Mitra 2010). Researchers argue that the greater leniency associated with the change in eligibility, the increased awareness of the programme and a rising prevalence of HIV and TB are potentially responsible for the increased access to the grant (CASE 2005; Mitra 2010). In this context, South Africa’s DG programme has been criticised for not being sustainable as it may provide disincentives in terms of job-seeking and health-seeking activities. This is based on the assumption that people hold off life-saving medications such as antiretroviral treatment for HIV and AIDS in order to be eligible for the DG (Kagee 2014; Nattrass 2005; Standing 2008). However, the latter is mostly based on anecdotes and not empirical evidence (Kagee 2014; Nattrass 2005; Phaswana-Mafuya, Peltzer & Petros 2009).

On the contrary, when using South African data from the General Household Survey (GHS) 2005 and Labour Force Survey (LFS) 2001–2003, Mitra (2010) found that although the DG reaches households that are poorer, have more children and higher unemployment rates, there is no evidence that it functions as a work disincentive. Her work shows that households were already detached from the labour market prior to the increased availability of the grant. Hence Mitra argues that in principle the DG is well placed to target poor households, but that its current application still involves both exclusion and inclusion errors. Based on her calculation, 34% of the DG beneficiaries were receiving the grant without work-related disability^1^ (inclusion error), while 42% of people who might be eligible (because of work disability and income status) did not receive the grant (exclusion error). This provides serious questions with respect to the eligibility determination process regarding who gets the grant and why. In addition, Mitra explored the effects of the 2001 change in policy revealing that the modifications to the assessment process increased access to the grant but did not alter labour market participation. Hence, the change in policy improved the accessibility of the DG, and the evidence suggests that the grant provides no work disincentive to those who might otherwise be seeking work. Similar assessments are not available on other disability-related grants such as the CDG and the GIA. The CDG is designed for caregivers of children with disabilities, and the GIA targets people who already receive a grant but have severe disabilities requiring full time care. The GIA is accessed by a relatively small part of the population, and its value is fairly small (e.g. R250 in 2011) (SASSA 2013a).

The literature highlights the importance of the DG eligibility criteria and the determination process with regard to exclusion and inclusion errors. However, there is a little understanding of who among the diverse group of people with disabilities is in need of social protection and who is lacking access to these mechanisms. One of the few articles looking into this question describes the uptake of the DG in a group of Xhosa speaking people in South Africa (Jelsma et al. 2008).The authors reveal that people with more severe disabilities were more likely to receive the grant and that people with ‘less visible conditions’ such as pain or loss of mental functions were less likely to receive the grant. This highlights the importance of understanding the nuances of disability to successfully direct the scarce resources (social grants) in MIC to reach those who are in need. However, to date very little is understood on how and when diverse disabling conditions are linked to economic vulnerability, and how social protection mechanisms can counteract these vulnerabilities most efficiently in MICs such as South Africa.

## Data and methodology

This article attempts to describe elements of the economic vulnerability of individuals and households with disabilities in South Africa. The 2011 GHS includes a set of disability-related questions as well as socioeconomic questions, making an analysis of disability and economic indicators possible. The rationale for using the 2011 version of the GHS is based on the comparability possibilities with the South African Census 2011. The Census while suited to the analysis of prevalence estimates does not contain as detailed information on the labour market as the GHS. The paper investigates elements of vulnerability on an individual and household level, as well as the impact of cash transfer programmes in compensating for this vulnerability. Based on the above, our first hypothesis is that disability is associated with economic vulnerability in the form of income loss, as well as multidimensional poverty in the form of lack of education and employment opportunities. Our second hypothesis is that this level of economic vulnerability varies in accordance with gender, degree and type of disability.

This article uses a model of disability-driven economic vulnerability developed by Hanass-Hancock and Deghaye in 2014, as a guiding framework (South African Department of Social Development 2016). In this model, economic vulnerability is understood to be created through disability-related opportunity costs (lack of education, employment opportunities, days out of role) and out-of-pocket costs (increased cost of healthcare, assistive devices and support, transport, etc.) that both influence available household income negatively. The model also highlights that this can be compensated through social protection mechanisms such as grants, free or affordable access to healthcare, education and other services, as well as disability-sensitive tax rebate systems.

In using the GHS 2011, this article aims to identify the potential opportunity costs of disability and the impact of grants at both individual and household levels. The GHS is a nationally representative household survey that is conducted annually to measure the level of development and performance of various government programmes and projects (Stats SA 2012). It collects socio-demographic information (gender, race, age, education, employment), contains questions on health and disability and access to social grants and basic services (housing, water, sanitation, electricity, refuse removal, transport, health and food supplies) as well as income (including source of income) for each individual.

The GHS captures information on disability through the use of the Washington Group (WG) short set of questions (Box 1). These questions collect information regarding any difficulties associated with seeing, hearing, communicating, mobility, concentrating or remembering and self-care even when using assistive devices. The WG short set of questions is a validated set of six questions that measures functioning in these six domains on a four-point Likert scale ranging from ‘no difficulties’ to ‘cannot do at all’. The WG has developed and tested these questions and found them robust in several countries including LMIC (Loeb 2012; Loeb, Eide & Mont 2008). Despite this rigorous testing, the WG short set of questions has been subject to some criticism. Two recent studies conducted in Cameroon and India have found that up to 46% of the people identified as having a disability through clinical screening methods were missed by the WG set of questions [International Centre for Evidence in Disability (ICED) 2014a, 2014b]. The short set of questions does not necessarily detect people with upper body mobility problems, intellectual disabilities or mental health disorders. In addition, statistical evaluation often uses a disability index method to establish disability prevalence, in the attempt to exclude all people with mild forms of functional limitations that may not actually be disabilities. However, this method could inadvertently be excluding people with disabilities thereby undercounting disability prevalence (Statistics South Africa 2014). Besides these limitations, to date this set of questions is believed to be the most accurate measure that can be included in population-based surveys.

BOX 1 Washington Group short set of questions on disability as used in the General Household Survey 2011.

**Does anyone have difficulty in doing any of the following**:

- seeing (even with glasses),
- hearing (even with a hearing aid),
- walking a kilometre or climbing a flight of stairs,
- remembering and concentrating,
- self-care or
- communicating (in their own language, including sign language).

Individuals are asked to rank their difficulties as follows: no difficulty (1), some difficulty (2), a lot of difficulty (3), or unable to do (4).

*Source:* GHS 2011 Survey Tool

The analysis that follows is predominately descriptive focusing on adults of working age, 15–59 years. We assess the impact of disability at the household and individual levels. For the purposes of this paper, taking into account the limitations of the questions asked in the GHS, the WG short set of questions was used to identify households and individuals with disabilities. Individuals with disabilities are identified when the survey respondent answered yes to at least one of the WG short set of questions. A household with disabilities was identified as having at least one member with a disability. The educational attainment, employment status and income of individuals are analysed to establish potential opportunity costs of disability. The potential economic impact of disability on the household is examined in terms of household access to income and social security grants. In both the analysis of the individual and household, we look at the earned income from employment, income from other sources and social grants. The DG is separated out from all of the other grants, as it is the most frequently accessed and is the largest grant that specifically targets people with disabilities who are of working age.

Disability is defined in terms of two degrees of severity. Firstly, ‘all degrees of disability’ represents a broad measure of disability, and this is then narrowed down in terms of only capturing severe disabilities as identified by the survey questions. ‘All degrees of disability’ accounts for all those individuals indicating they have at least some difficulty doing at least one of the activities listed in the WG short set of questions (Box 1). These activities range from physical and sensory to more cognitive activities. Severe disability refers only to those individuals with a lot of difficulty or those unable to perform any one of these activities. It should be noted that the group including ‘all degrees of disability’ ranges from mild to severe disabilities while severe disability only captures those with more severe difficulties; thus, the two definitions are not mutually exclusive. The economic outcomes and exposure to social protection measures are assessed for people with disabilities based on the aforementioned definitions. In addition, we include a disaggregated analysis of disability in this paper, highlighting the difference in outcomes for people and households with regard to the six different disability types measured in the WG set of questions.

## Results

### Individuals

The overall prevalence of disability in South Africa is 12% based on the 2011 GHS. This includes individuals aged 6 years and older. Of this, just over 3% indicate a severe disability. The majority of people with disabilities are of working age as just over 50% of those with all degrees of disability, and nearly 40% of those with severe disabilities, are aged 15–59 years. The analysis that follows therefore deals specifically with the working age population.

Table 2 presents descriptive statistics showing the prevalence of disability by gender, employment status and earnings. All figures are statistically significant at the 5% level indicating that groups of individuals with disabilities (all or severe) are significantly different from those with no disabilities. Income data are based on reported earnings from employment, and the mean estimates are presented here. Grant income is separated into two categories to highlight the DG. The category ‘Other Grants’ includes the OAG, CDG, GIA, CSG, FCG and WVG. In all cases, we present the average income (from employment or grants) for a particular subset of the population.

**TABLE 2:** Individual characteristics of people aged 15–59 years.

Characteristics	All degrees of disabilities % (SE)	Severe disabilities % (SE)	No disabilities % (SE)
Gender (%)			
Male	42.9* (0.8)	48.9 (1.7)	48.9 (0.3)
Female	57.1* (0.8)	51.1 (1.7)	51.1 (0.3)
Mean number of years of schooling	8.66*	6.65*	9.87
Employment status (%)			
Inactive	36.5* (0.8)	61.8* (1.6)	26.2 (0.2)
Unemployed and not searching for work	4.2* (0.3)	4.4 (0.7)	5.4 (0.1)
Unemployed and searching for work	18.6* (0.6)	13.7* (1.1)	24.8 (0.2)
Employed	40.7* (0.8)	20.1* (1.3)	43.6 (0.3)
Average monthly income from employment (in rands)	7272.55* (332.1)	4645.30* (470.37)	6009.30 (107.0)
Average grant income incl. disability grant (in rands, per month)	205.71* (6.6)	497.20* (18.9)	40.11 (0.9)
Average other grant income excl. disability grant (in rands, per month)	21.97 (2.1)	26.74 (5.7)	20.19 (0.6)

*Source:* GHS 2011, own calculations

Those individuals with severe disabilities are also captured in the ‘all degrees of disabilities’ category. The data have been weighted to represent the South African population. The sample consists of adults aged 15–59 years. Standard errors (SE) are in parenthesis.

* , represents significance at the 5% level and is with respect to the ‘no disabilities’ category in each case.

The average Rand–US dollar exchange rate for 2011 was R7.25 per USD 1.

On the whole, women are slightly more likely to report disabilities compared with men. The employment rates for individuals with disabilities are generally lower compared with those with no disabilities, particularly in the group with severe disabilities where only 20% of individuals are employed (Table 2). In addition, the data revealed that those with disabilities are more likely to remain out of the labour force compared with individuals with no disabilities. A significantly higher level of inactivity was noted among those with severe disabilities: almost two-thirds of individuals are economically inactive. This may be linked to the likelihood that more severe disabilities can limit an individual’s job prospects thus causing them to remain out of the labour force. People with disabilities also have significantly fewer years of education on average compared to people with no disabilities, which again limits job prospects. In our analysis, people aged 15–59 years with no disabilities have close to 10 years of education on average, whereas those with severe disabilities have at least a third less. Not surprisingly the average monthly earned income from employment for people with severe disabilities is R4645.00, which is much lower compared to people without disabilities.

However, employed individuals with any degree of disability do not appear to experience lower income levels compared with those individuals without disabilities. On the contrary, this group emerged as higher earners. This may seem contradictory to the conventional wisdom of negative associations between disability status and income. However, upon closer investigation, results show that this positive association could potentially be a result of the younger average age of those without disabilities (Table 3). Typically, younger individuals starting their careers earn less than those who have been working for a number of years in relatively established careers (Becker 1962). In addition, a large portion of individuals with mild visual disabilities were in this sample, and these were identified as the higher earners.

**TABLE 3:** Average age of employed individuals aged 15–59 years.

Variable	All degrees of disabilities	Severe disabilities	No disabilities
Average age of employed individuals (years)	46*	47*	38

*Source:* GHS 2011, own calculations.

Those individuals with severe disabilities are also captured in the ‘all degrees of disabilities’ category. The data have been weighted to represent the South African population. The sample consists of households with adults aged 15–59 years.

* , represents significance at the 5% level and is with respect to the ‘no disabilities’ category in each case.

## Households

Indications of economic vulnerability are also noted at the household level. Household characteristics presented in Table 4 illustrate a comparison between households with and those without disabilities. All figures are statistically significant at the 5% level with regard to households with no disabilities, showing that households with disabilities are statistically different from those without. Household income was calculated by summing the earnings of all employed household members, and we present the average household monthly earnings from employment for our three subsets of the population. Other sources of income refer to rental income and any income that is not earned through employment. Once again, income from grants is separated out into two categories to highlight the DG on its own. All of these sources of income summed together provide the figures for the average total monthly household income (including grants).

**TABLE 4:** Household characteristics.

Characteristics	HH with at least one person with disabilities (any degree)	HH with at least one person with severe disabilities	HH with no disabilities
Geographical location			
Urban	64.0* (0.90)	57.2* (1.70)	66.5 (0.40)
Rural	36.0* (0.90)	42.8* (1.70)	33.5 (0.40)
Mean number of household members employed	1.06* (0.02)	0.75* (0.04)	1.18 (0.01)
Household average monthly income from all sources:			
Employment (in rands)	6020.55* (324.80)	3369.30* (375.40)	5454.20 (131.00)
Other sources (in rands)	291.80* (23.60)	274.70* (34.60)	415.40 (18.90)
Disability Grant (in rands)	282.50* (10.60)	617.30* (28.70)	54.82 (2.07)
Other grant income (in rands)	535.45* (14.00)	723.23* (30.35)	497.70 (6.50)
Total grant income (in rands)	817.90* (19.40)	1340.50* (45.80)	552.50 (7.04)
Total income (including grant income) (in rands)	7130.23* (322.20)	4984.40* (364.20)	6422.10 (130.81)

*Source:* GHS 2011, own calculations

Those individuals with severe disabilities are also captured in the ‘all degrees of disabilities’ category. The data have been weighted to represent the entire population of South Africa. The sample consists of households with adults aged 15–59 years. Standard errors (SE) are in parenthesis.

* , represents significance at the 5% level and is with respect to the ‘no disabilities’ category in each case. The average Rand–US dollar exchange rate for 2011 was R7.25 per USD 1.

HH, Households.

Table 4 indicates that the majority of households with at least one individual with any degree of disability reside in urban areas compared to households with no disabilities. However, there are proportionately more households with at least one person with severe disabilities in rural areas. The household size is smaller if there is an individual with severe disabilities living in the household.

On average, households with disabilities (any degree) seem to earn more than households with no disabilities. This is because of both income from employment and grant income being significantly higher in comparison. As indicated in Table 3, the average age of individuals with disabilities is 46 years (all degrees of disability) which may imply that some of these more mild disabilities relate to the normal ageing process or disease progression. Households with at least one individual with severe disabilities, however, earn significantly less compared to both the group including all degrees of disabilities and households with no disabilities. While grant income does succeed somewhat in closing this gap, it is still not enough to enable households with severe disabilities to reach the same income level as those with no disabilities. Thus, households with severe disabilities are more vulnerable to poverty.

**FIGURE 2:**
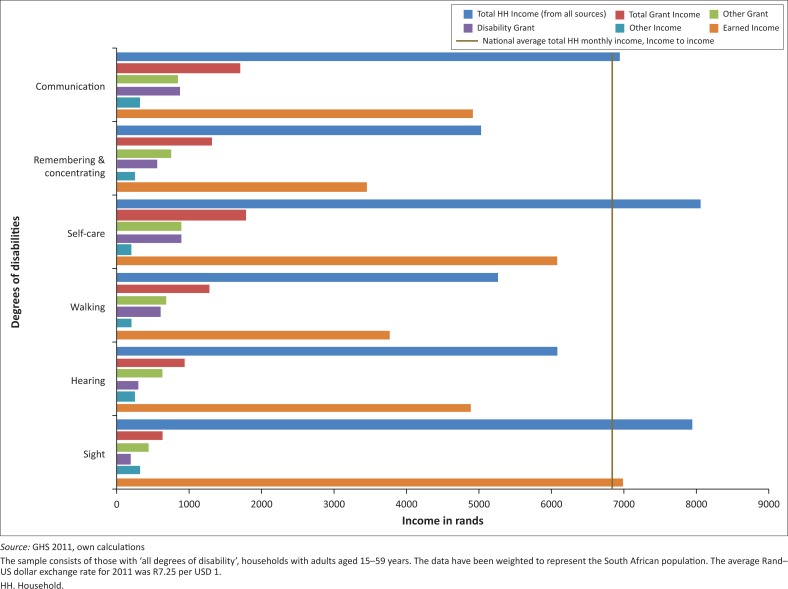
Average monthly household income, adults 15–59 years with all degrees of disabilities, by type.

**FIGURE 3:**
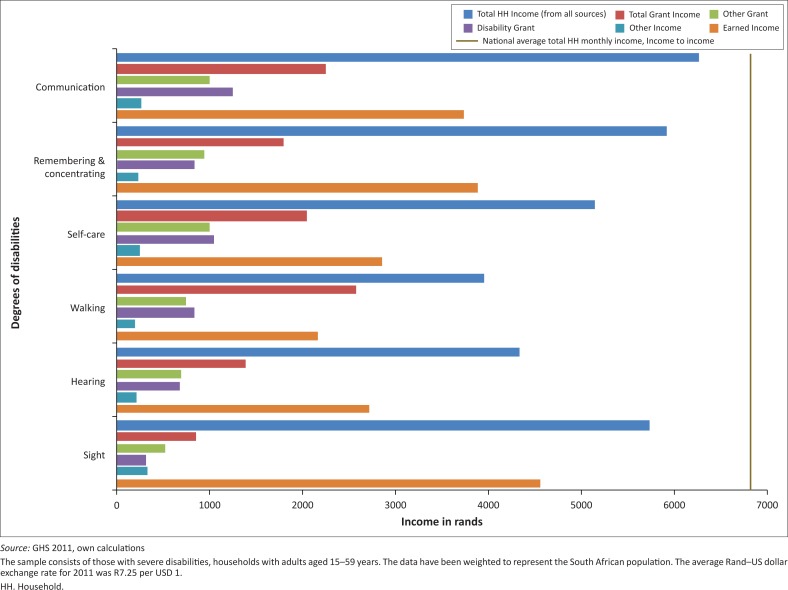
Average monthly household income, adults 15–59 years with severe disabilities, by type.

To offer a more in-depth look at the incomes of households with people with disabilities, data were disaggregated by disability type. There are six categories as provided by the survey questions: sight, hearing, walking, remembering and concentrating, communication and self-care. Figures 2 and 3 illustrate income from all sources by disability type. The national average household income is R6800 per month; households with all degrees of disabilities typically earn below that except for those with sight difficulties (Figure 2). The groups who appear to be most economically vulnerable are those with walking, hearing and remembering and concentrating difficulties.

For households with people with severe disabilities, income is substantially lower in the case of all disability types (Figure 3). The addition of grant income does not succeed in pushing income levels anywhere near the national average. The groups who are relatively worse off are households with at least one individual with severe difficulties hearing, walking and with self-care. These households have, on average, the lowest earned income, which refers to income from employment. In most instances, households including people with any degree of disability have an overall higher level of income compared to those with severe disabilities. However, in the category referring to difficulties remembering and concentrating (Figure 3), households with any degree of this disability type earn on average less than those with severe disabilities. The data show that this higher income is driven by both income from employment and increased access to social grants.

## Discussion

Social protection mechanisms are a new way of addressing structural inequalities in MICs such as South Africa. It is therefore important to assess how these mechanisms currently reach those in need and how they can be better used to compensate for any inequalities that may exist. The core interest of these mechanisms should lie within the equalisation of opportunities through equitable rather than equal social protection mechanisms.

Using the GHS and the WG short set of questions, this study is limited by the disability types and economic questions that were asked in this survey. Hence, it only covers some disability types and only elements of economic vulnerability. Nevertheless, the study highlights that in the context of disability, careful consideration must be given to the diverse, nuanced nature of disability as it relates to type and degree of disability as well as age. Previous work has highlighted that the association between disability and characteristics such as gender, geographical location and race multiplies vulnerabilities (Stats SA 2014). Stats SA (2014) has already provided strong evidence of the double burden women with disabilities face, where women with disabilities are less likely to be employed and if they are employed, they are typically low earners. This article takes the analysis further and discusses the complexity of disability in relation to the degree and type of disability showing how these factors impact economic vulnerability in terms of income, education and employment. It is now a matter of urgency to determine the best possible approach to account for these complexities while developing social protection mechanisms in resource-poor settings. This should be done in a feasible manner without complicating the process of eligibility and assessment.

This study illustrates that people with all degrees of disabilities have fewer years of education and are less likely to be employed compared to those with no disabilities. People with severe disabilities in particular are more inclined to be out of the labour force altogether. Of those employed, people with all degrees of disabilities earn more on average compared to those with no disabilities. This would seem to contradict the theory, which suggests that people with disabilities are worse off financially relative to people with no disabilities. However, given that this particular group of people includes those with both mild and severe disabilities, and the fact that South Africa has progressive labour laws, it is possible that milder disabilities are less affected by socio-economic difficulties. This finding is similar at the household level. Further examination revealed specifically that households with people with difficulties seeing (any degree of disabilities) had average incomes comparable to households without people with disabilities. This is largely because of higher earned income (from employment) for this group. Given that a large number of these individuals are between the ages of 40 and 59, it is possible that the income results reflect people who acquired visual impairments through normal ageing or disease progression while already being better earners with prior-established careers. Milder disabilities, as captured under ‘all degrees of disability’, are also potentially less likely to rely on caregiving as by definition the person has only some difficulty performing an activity (see survey question, Box 1). This could then result in less strain on the household in terms of earnings and fewer household opportunity costs in terms of a loss of employment or earnings because of caregiving.

In terms of the other disability types, all households (any degree or severe) have considerably less income than households without people with disabilities and lie far behind the national average. Given that this is household income, it implies that relatively fewer household members are working or that those who are working are in low-paying jobs. It is possible that a large portion of individuals with severe hearing, walking or self-care difficulties are actually unemployed and may even require care from another household member thus reducing the income potential for that household. Social grants do go some way to assist these households, but they are still worse off overall in terms of total average monthly income from all sources. The severe category refers to individuals who have a lot of difficulty or cannot perform a specific task at all. In this case, it is more likely that individuals are unemployed and require care or assistance which means that other household members may have to forgo their own employment. Fewer opportunities for earnings result in households with severe disabilities earning just over half the average monthly income of households within the ‘all degrees of disabilities’ category and households with no disabilities.

Social protection mechanisms in the form of grants have different effects on each group. These grants are equally distributed among all groups; however they are less equitable and do not compensate for the different degrees of economic vulnerability associated with different types and severity of disability. Grants only seem to compensate (in terms of bringing total household income somewhat closer to the national average) those households that have people with communication problems. In all other sub-groups this level of compensation has not been reached. However, for some households with people with severe disabilities (hearing, walking) grants almost double household income, which is a considerable achievement for a MIC like South Africa. For most disability types the overall income of households in the severe disability group is lower than that of those with all degrees of disabilities with the exception of households with people with difficulties remembering and concentrating. In the latter group those households with people with severe disabilities have on average, a higher overall income. This is potentially because of a greater effect of social grants for this cohort or the possibility of measurement error associated with over or under reporting for both mild and severe difficulties.

Although this analysis for South Africa suggests that social grants partially compensate income losses for those households that have people with disabilities (and for some households even up to the level of the average national monthly income), the substantial disability-related out-of-pocket costs are not considered in this data (International Labour Office & International Disability Alliance 2015; Palmer et al. 2015). These out-of-pockets costs have been described in an earlier study (Banks & Polack 2013), as well as in an upcoming publication (Hanass-Hancock et al. 2017). These costs are diverse and can be very high for some groups. Therefore, social protection mechanisms need to be designed more equitably and respond to the care and support needs rather than just the identification of disability status. This applies to the DG as well as to the GIA and CDG, which are both designed to compensate costs related to increased care. However, the GIA is not significant to cover the care and support for people ‘who need full time attendance by another person’ (SASSA 2013b) (R250 per month in 2011 and R350 in 2017) which would only realistically cover a caregiver for a few days.

The results also indicate that in some cases those in the ‘all degrees of disabilities’ group are less likely to access the DG and this applies in particular to the group of people in the category of remembering and concentrating. This may be an exclusion error related to the eligibility determination process as this type of ‘disability’ is less visible and less likely to be identified or people may be reluctant to be identified as ‘disabled’. These challenges could apply to people with mild intellectual impairments, people with mental health problems, older people or people from the autistic spectrum. Social protection mechanisms that also target people with less ‘visible’ disabilities are therefore needed, and, consequently, the current grant assessment procedures may have to be reviewed. The DG assessment process and criteria have not seen any significant changes since its introduction in 1946, except for that described by Mitra (2010). Ideally, these assessment procedures should follow a targeted, nuanced approach which may involve a more complex process of establishing eligibility for all disability-related grants.

This study also poses a question with regard to the purpose of the DG. Although the analysis reveals that social grants compensate income losses for those households with people with disabilities, it remains to be shown if the DG is a poverty grant or a grant that tries to provide social assistance to people with disabilities. The two approaches are quite different; while the first has the underlying assumption that people with disabilities are economically vulnerable and less able to participate in the labour market, the latter asks what types of social support enables people with disabilities to participate on an equal basis in society including the labour market. While the former can function as a charity hand-out and may even be seen as a ‘poverty trap’, the latter asks what kind of social assistance will enable participation and independent living (hence is Convention on the Rights of Persons with Disabilities, CRPD, compliant).

**FIGURE 4:**
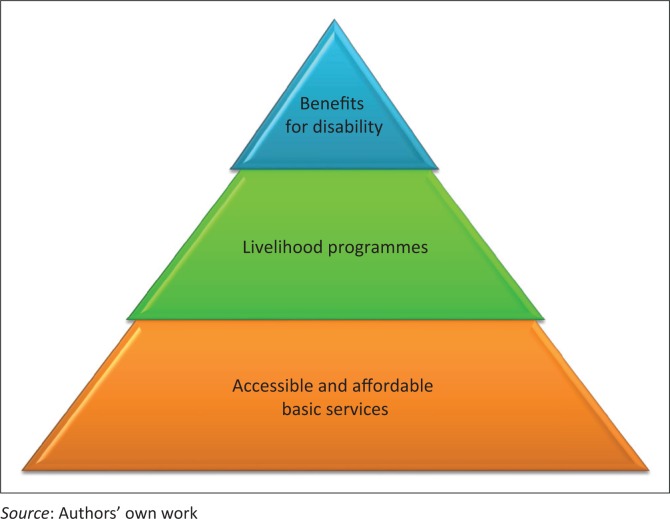
Triple social protection mechanisms to ensure participation of people with disabilities.

To answer the latter, one would have to understand two elements. Firstly, the extra costs associated with disability need to be identified which include not only opportunity costs but also costs of disability accommodation, assistive devices, care and support, housing and additional healthcare needs. Secondly, the physical and social barriers that people with disabilities experience in all aspects of life need to be identified and removed. This in particular applies to access to transport, buildings, communications and information. Ideally social protection mechanisms would then consist of three elements (Figure 4): accessible and affordable basic services (health, education, transport), mainstream livelihood programmes including people with disabilities (cash transfers, microcredits, housing, insurance, workplace protection) as well as targeted support for people with disabilities in order to address their households’ disability-related costs (opportunity or out-of-pocket).

To be equitable these disability benefits need to be measured on the basis of the care and support needs of people with disabilities and not on the basis of fulfilling general disability-eligibility criteria. Currently, only people with disabilities who are deemed unfit to work actually qualify for the DG subject to a means test. The DG should become more multifaceted such that people are given sufficient financial support to encourage labour market participation. In addition, if people with disabilities are subject to a means test, the threshold should be higher than that of other poverty-related benefits. This is because disability-related costs remain constant or may increase once a person is able to participate in the labour market (Hanass-Hancock et al. 2017).

The new South African White paper (South African Department of Social Development 2015) particularly focuses on overcoming economic vulnerability for persons with disabilities. It includes an ambitious operational plan and covers all three aspects highlighted in Figure 4. The next 5 years will show the extent to which South Africa will be able to implement these ambitious targets. To achieve this, South Africa needs to enforce a number of its already progressive policies, push towards better accessibility of crucial services (education, health, transport) and monitor its progress towards this new set of goals. Attention needs to be paid to the current gaps within the system, which includes access to schooling and employment for specific groups of people with disabilities. Currently, we do not understand the resources and cost allocations needed to develop inclusive education, health and supporting services (e.g. transport) such that we reduce the economic vulnerability of people with disabilities (South African Department of Social Development 2016). Research in this area is urgently needed for South Africa and other LMICs.

Furthermore, our data and other literature suggest that people with mild or less visible disabilities are being excluded from disability benefit schemes and families are sacrificing their own employment and earnings prospects because of caregiving duties as a result of the current lack of state care and support. Both mainstream as well as disability-focused research have a key role in describing these opportunity and out-of-pocket costs for this part of the population and provide better evidence on how to design disability inclusive social protection mechanisms that are feasible for countries such as South Africa. South Africa still has a long way to go to adjust current social protection mechanisms so that they are addressing disability-related economic vulnerability. Data and research that can inform this process are still emerging in the country.

The experiences from South Africa can also inform current efforts by international agencies that are moving towards the inclusion of people with disabilities in the new developmental strategies that target poverty alleviation and social protection in a number of LMICs (ICED 2014b; International Labour Office & International Disability Alliance 2015). Agencies such as the International Labour Office and International Disability Alliance are already developing a common understanding of the nuanced nature of the disability-poverty nexus, as well as strategies of appropriate and disability-specific practices in terms of social protection that are in line with the CRPD (International Labour Office & International Disability Alliance 2015). The complexity of this undertaking has been acknowledged, and the experience from South Africa provides important data to inform the process of developing disability-specific social protection mechanisms in other countries (International Labour Office & International Disability Alliance 2015).

## Footnotes

1 People who cannot work because of disability.
